# Multi-subunit BCG booster vaccine GamTBvac: Assessment of immunogenicity and protective efficacy in murine and guinea pig TB models

**DOI:** 10.1371/journal.pone.0176784

**Published:** 2017-04-28

**Authors:** A. P. Tkachuk, V. A. Gushchin, V. D. Potapov, A. V. Demidenko, V. G. Lunin, A. L. Gintsburg

**Affiliations:** 1 Translational Biomedicine Laboratory, N.F. Gamaleya Federal Research Centre for Epidemiology and Microbiology, Moscow, Russia; 2 A.N. Belozersky Institute of Physico-Chemical Biology, Moscow State University, Moscow, Russia; 3 State Research Center for Applied Microbiology and Biotechnology, Obolensk, Russia; 4 Laboratory of bioactive nanostructures, N.F. Gamaleya Federal Research Centre for Epidemiology and Microbiology, Moscow, Russia; 5 N.F. Gamaleya Federal Research Centre for Epidemiology and Microbiology, Moscow, Russia; Fundació Institut d’Investigació en Ciències de la Salut Germans Trias i Pujol, Universitat Autònoma de Barcelona, SPAIN

## Abstract

New innovative vaccines are highly needed to combat the global threat posed by tuberculosis. Efficient components–antigens and adjuvants–are crucial for development of modern recombinant TB vaccines. This study describes a new vaccine (GamTBvac) consisting of two mycobacterial antigen fusions (Ag85A and ESAT6-CFP10)–with dextran-binding domain immobilized on dextran and mixed with an adjuvant consisting of DEAE-dextran core, and with CpG oligodeoxynucleotides (TLR9 agonists). GamTBvac and its components were assessed for immunogenicity and protective efficacy in GamTBvac-prime/boost and BCG-prime/ GamTBvac-boost in murine and guinea pig TB models. Results show that in both infectious models, GamTBvac has a strong immunogenicity and significant protective effect against *Mycobacterium tuberculosis* strain H37Rv under aerosol and intravenous challenges. GamTBvac showed a particularly strong protective effect as a BCG booster vaccine.

## Introduction

Despite great international advances, tuberculosis (TB) remains one of the world’s biggest threats. TB killed more than 1.4 million people (1 million HIV-negative and 0.4 million HIV-positive patients) in 2015 [1]. There are diagnostic methods and intensive treatment schemes designed and widely implemented to reduce TB mortality. However, the results have not achieved expected targets [2,3]. HIV epidemic is assumed to be the driving force behind the spread of multidrug resistant tuberculosis (MDR-TB) and extensively drug-resistant tuberculosis (XDR-TB). It is also regarded as the cause of reactivation of latent TB infection (LTBI) [3]. On one hand, this situation is complicated by the slow progress in developing new chemotherapeutic agents against resistant strains, and on the other hand—by the fact that over two billion people worldwide today are latently infected with *M*. *tuberculosis* (MTB) [4].

Vaccination still remains the most cost-effective way of minimizing TB cases and deaths. However, the only recommended vaccine is the *Mycobacterium bovis* BCG (Bacillus Calmette-Guerin) introduced in 1921. BCG protects children against miliary TB and meningitis. However, this protection vanishes 10–15 years after the vaccination [5,6]. The BCG vaccine can be anywhere from 0 to 80% effective in protecting against pulmonary TB [7,8]. Although BCG revaccination is the national standard for Russia and Japan, its propriety is now doubtful. BCG revaccination is not found to be effective in animal TB models [9] and humans [10]. With the above-said, it is clear that new TB vaccines are highly needed. TB vaccine candidates should be able to induce persistent and specific immunity through activation of proper repertoire of T cells. The candidates should be safe, accessible and reliable because over 80% of all new cases occur in developing countries [2]. Besides, ideal TB vaccines should be effective in different groups of people (unvaccinated, BCG vaccinated, people infected with HIV and TB (including latent form), and patients that recovered from TB). The vaccines should also prevent latent TB reactivation.

About 13 TB vaccine candidates are currently under clinical trials (live whole-cell vaccines [recombinant BCG and attenuated *M*. *tuberculosis*], proteins with an adjuvant, virally vectored proteins and mycobacterial lysates) [4]. Most of them are aimed at enhancing BCG-induced immunity—the so-called BCG booster vaccines [11]. The target of such vaccines is to refresh the BCG-induced immunity but not to achieve sterile eradication or prevention of stable infection [4]. The boosters are based on one or more selected MTB antigens expressed by a live viral vector or produced as recombinant proteins. Recombinant protein composition has a number of production advantages, such as cost effectiveness, easy scalability, high level of safety, and many simple quality control parameters for full characterization [12].

Antigenic composition is a determinant in recombinant vaccine development [4]. Most subunit vaccine candidates are based on one to four antigens, defined using traditional biochemical methods. Such antigens are usually recognized by T cells from LTBI patients or patients cured from TB. Immunogenicity and immunodominance are important indicators for antigen selection [13]. Synthetic peptide mixes facilitate the testing of putative vaccine antigens in a high throughput manner [13]. Most of the known antigens that are part of the TB vaccines being developed, as well as interferon gamma release assays (IGRA), were shown to be very immunogenic and immunodominant. It is known now that multiple, multistage antigens give better protection, and work better than separate component mixtures when fused in frame antigens [14,15]. The main problem is that proteins are not inherently immunogenic and need adjuvants to strengthen the immune response to vaccine.

An effective recombinant TB vaccine would require an effective and safe adjuvant that is capable of inducing proper polarisation of cellular immunity (for review see [12]). Adjuvants approved for human vaccines, including alum and oil-in-water based emulsions MF59 (Novartis Vaccines and Diagnostics), AS03 (GlaxoSmith Kline Biologics, GSK), AF03 (Sanofi), and liposomes (Crucell) are effective in inducing primary humoral responses but not less effective in activation of cell-mediated immunity (CMI) [16]. There is no evidence that these adjuvants induce CMI response needed for combating an intracellular pathogen like *M*. *tuberculosis* [12]. It was shown that TLR agonist additives improve adjuvant performance [17]. For a range of new adjuvants that induce CMI response (CAF01, GLA-SE and IC31) distinct antigen-independent immunological signatures tailored to different pathogens were shown [18]. Thus immune response polarisation is significantly modulated by adjuvant. It is generally recognized that CMI Th1 response is determinant of TB protection [18]. Modern TB adjuvants are two-component in nature—combining both a delivery system and an appropriate immunomodulatory effect [12]. An ideal adjuvant delivery system should be able to enhance antigen uptake and presentation, protect antigen from degradation, direct stimulation towards the desired immunological path, suppress the toxicity of PAMPs (pathogen associated molecular patterns) systems and slow down release of antigen from the carrier to prolong antigen specific stimulation.

Dextran is one of the most investigated α-glucans in drug and antigen delivery (for review see [18]). It is a cheap reagent, has a long history of medical usage and is generally recognized as safe (GRAS) by the FDA. Dextrans are approved for use as biodegradable materials in surgery, for detoxification and correction of rheological properties of blood. Dextran by itself is immunogenic and can induce both humoral and cell mediated immunity [19]. Dextran can directly interact with cellular receptors mediating phagocytosis DC-SIGN. In combination with dextran-DEAE (a polycationic derivative of dextran), it could be a promising smart adjuvant delivery system supporting deposition of both mixtures of recombinant antigens fused with dextran-binding domains and mix of CpG oligodeoxynucleotides TLR9 agonists [12,19,20,21].

So combination of proper set of antigens in fusion, adjuvant properties and concentration of vaccine components are important for necessary protective immunity [2,12]. In this study, we have explored and tested a vaccine made of two MTB antigens (Ag85A and ESAT6-CFP10 in frame fused with dextran-binding domain (DBD) from *Leuconostoc mesenteroides* [21]) non-covalently immobilized on dextran. Nanoparticles consisting of DEAE-dextran core—covered with a mixture of CpG oligodeoxynucleotides (TLR9 agonists)–were used as the adjuvant. This combination in GamTBvac vaccine was experimentally assessed for immunogenicity and protective efficacy in GamTBvac-prime/boost and BCG-prime/ GamTBvac-boost regimens in mouse and guinea pig TB models.

## Materials and methods

### Ethics statement

Procedures involving animals were performed in compliance with international recommendations Directive 2010/63/EU and Appendix A to the European Convention ETS No. 123. All mice and guinea pig experimental procedures were approved by the Local Animal Care and Use Committee of the N.F. Gamaleya Federal Research Centre for Epidemiology and Microbiology (GFRCEM). The animals were kept at an animal facility under specific pathogen free conditions. They received access to water and food *ad libitum* throughout the study. For *M*. *tuberculosis* H37Rv challenge experiments, the animals were kept in ABSL-3 lab at GFRCEM. Humane endpoints during the animal survival study were used. The animals were euthanized in a CO_2_ chamber or by cervical dislocation.

### Bacterial strains

For vaccination experiments, the BCG Russia 1 strain (Medgamal, Russia) was used. *M*. *tuberculosis* H37Rv strain (ATCC 93009) was prepared by the BSL-3 Lab at GFRCEM. *Mycobacterium tuberculosis* H37Rv was grown in Middlebrook 7H11 medium supplemented with glycerol (0.25% v/v) and L-asparagine (0.05% m/v). Bacterial suspensions were frozen and stored at -80°C. Serial dilutions of the bacterial suspensions were plated on 7H11 agar plates for colony-forming units (CFU) counts before use.

### Animals

Mice—female C57BL/6 mice (6–8 weeks old) were purchased from Pushchino Ltd., an animal nursery laboratory in Pushchino, Russia, and were kept under specific-pathogen-free conditions at GFRCEM. During the experiments, the mice were observed for clinical symptoms and weighed every day. Mice discovered to have critical ill symptoms (generalized intoxication, paresis, paralysis) or to be losing 1.5 g or more in weight every 3 days or more were euthanized.

Guinea pigs were purchased from the Andreevka Research Centre for Biomedical Technologies under the Russian Academy of Medical Sciences. Groups of male animals (255.6±4.6 g.) were acclimated for 5 days before the experiments. After infection, the animals were weighed. Guinea pigs were weighed every day and were euthanized based on 20% weight loss compared to maximum weight and increased respiratory rate (labored or heavy breathing). The general behavior and appearance were assessed by a veterinary specialist.

### Cloning and expression of recombinant proteins

**Fig 1:**
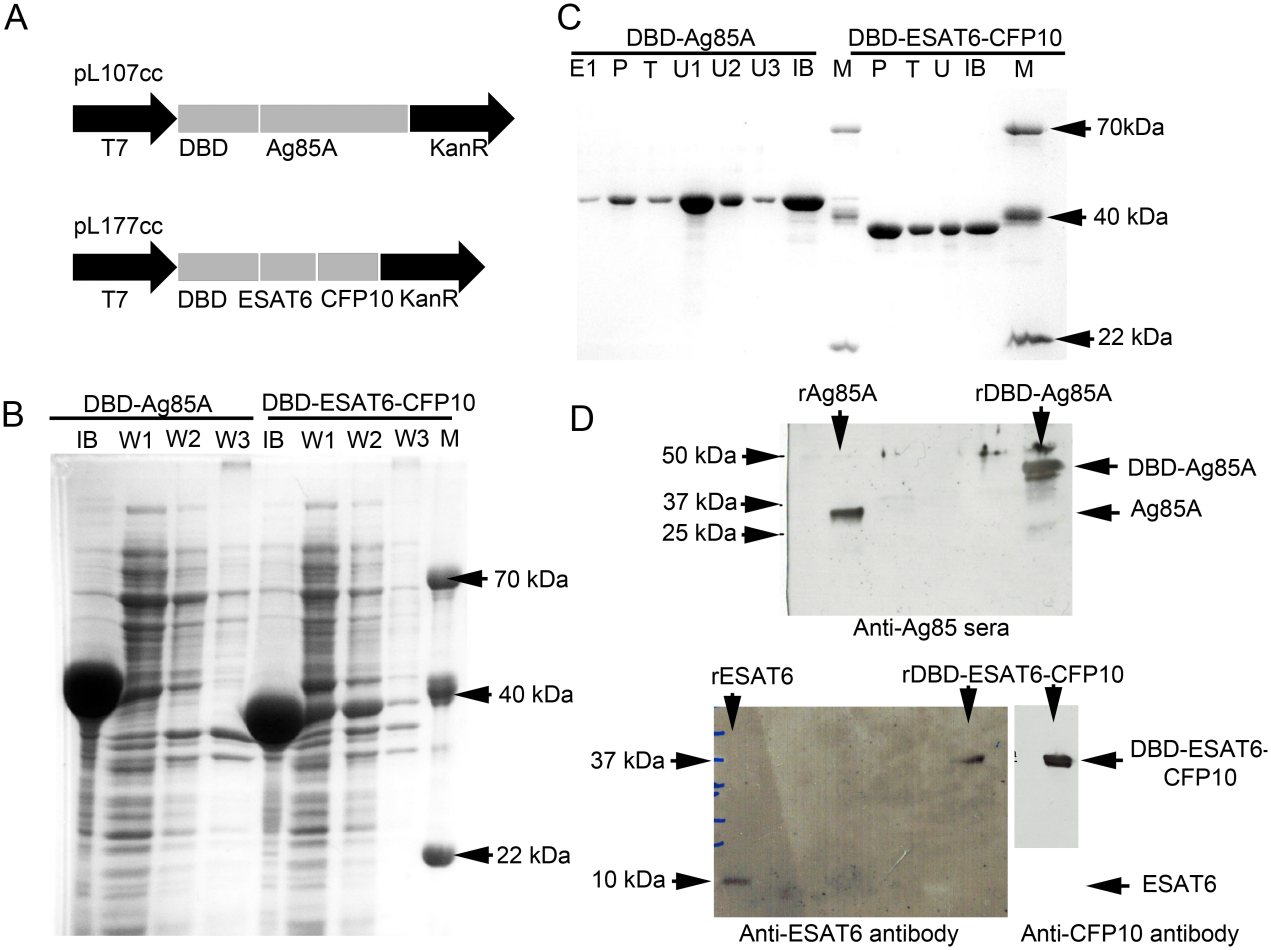
Expression and purification of GamTBvac components. Two antigen fusion proteins DBD-ESAT6-CFP10 and DBD-Ag85A **(A)** were induced by IPTG in *E*. *coli* BL21. Coomassie blue staining was used for evaluation of inclusion bodies washing results **(B)** and column purification process **(C)**; M—molecular weight marker, IB—inclusion bodies, W1-2 –wash of IB with 25 mM Tris pH 7.5 + 50 mM NaCl, W3 –wash of IB with 25 mM Tris pH 7.5 + 50 mM NaCl + 25%EtOH, U– 8M urea wash fractions, T—tail water elution fraction, P—peak water elution fraction, E—elution fractions. Both recombinant fusions were checked by western blotting **(D)** with rabbit antisera (for rAg85A) or antigen specific monoclonal antibodies (rESAT6 and rCFP10)

Synthetic genes corresponding to MTB antigen sequences Ag85a, ESAT6, CFP10 were cloned into the previously described prokaryotic expression vector pET28a, which includes dextran-binding domain (DBD) from *Leuconostoc mesenteroides* and Gly-Ser spacer [21,22]. As a result, two recombinant plasmids (DBD1-AG85a (pL107cc) and DBD1-ESAT6-CFP10 (pL177cc)) were constructed, each coding for a chimeric gene composed of nucleotide sequence of DBD gene, Gly-Ser spacer and nucleotide sequence of either Ag85a or ESAT6-CFP10 MTB antigens (Fig 1A). Sanger sequencing was used to verify DBD-Ag85A and DBD-ESAT6-CFP10. The recombinant plasmids were transformed into ClearColi^™^ *E*. *coli* BL21 (DE3) strain (Lucigen, USA, http://www.lucigen.com). Expression of target proteins was induced by incubation with isopropyl thio-β-D-galactoside (IPTG) at a final concentration of 1 mM for 6 hours. Recombinant proteins were purified from inclusion bodies in a buffer containing 8M urea, 25 mM Tris pH 7.5 and 50 mM NaCl. Inclusion bodies contained mainly target proteins (Fig 1B). Proteins were refolded in 25 mM Tris pH 8.5–8.8 and purified on the Butyl-Toyopearl hydrophobic column (Tosoh Bioscience LLC, USA). Proteins were verified by Western blotting against target proteins (Fig 1D). Purified proteins were lyophilized, diluted in phosphate-buffered saline (PBS) using pyrogen-free reagents, aliquoted and stored at -20°C. The absence of *E*. *coli* byproducts was confirmed by immunoblotting with horseradish peroxidase-conjugated rabbit polyclonal anti-*E*. *coli* antibody (1:1000; BiAlexa, Russia). Residual endotoxin contamination was evaluated using limulus amoebocyte lysate assay (LAL-test; Cambrex Corp., USA) and determined to be less than 16 endotoxin units per mg of protein. The vaccine was formulated with the adjuvant containing dextran 500kDa (10 mg/dose; Dextran 500 Pharmaceutical Quality, Pharmacosmos, Denmark), dextran DEAE 500kDa (0.5 mg/dose; DEAE-Dextran Pharmaceutical Quality, Dextran products limited, Canada) and CpG ODN 5’-ggGGGACGA:TCGTCgggggg-3’ (0.15 mg/dose; synthesized by the chemical group of the Laboratory of the Biologically Active Nanostructures at Gamaleya Federal Research Centre for Epidemiology and Microbiology) The final product (vaccine) was manufactured by Gamaleya Federal Research Centre for Epidemiology and Microbiology in an accredited GMP facility and supplied to the study site as a sterile suspension at pH 7.4 for injection.

### Immunization

Mice were injected with 0.5 ml of GamTBvac vaccine containing DBD-Ag85a and DBD-ESAT6-CFP10 (5, 10 or 20 μg/dose of each protein mixed or supplied separately). For this study, different formulations of recombinant vaccine GamTBvac were subcutaneously administered to the mice two times at 3-week intervals. BCG Russia 1 was used as a positive control and inoculated subcutaneously once with 5×10^6^ CFU during the first vaccination. PBS and other vaccine components (adjuvant, recombinant protein without adjuvant) were separately used as controls.

### Number of the T-cells producing IFNγ by ELISPOT

Five weeks after the last immunization, the levels of antigen-specific IFNγ in spleen and popliteal lymph nodes were analyzed. IFNγ was tested using an enzyme-linked immunospot (ELISPOT) assay following a 20-hour incubation of cells with specific antigens including DBD-Ag85a (10 μg/ml), DBD-ESAT6-CFP10 (10 μg/ml) and DBD (10 μg/ml). Binding IFNγ antibody (100 μL; 4 μg/ml in sterile PBS; clone R4-6A2, BD PharMingen) were incubated for 18–20 hours at +4°C in 96 well plates (Millipore, MAIP S45, USA). The plates were treated with 200 μL/well RPMI supplemented by 5% FCS at 37°C for 2–4 hours. Later, the cells in doublets (0.5–8×10^5^/well in 200 μL RPMI supplemented with 5% FCS; 5% CO_2_) with antigen (10 μg/ml) were incubated for 48 hours at 37°C. The plates were washed 5 times with PBS (200 μL/well) and once with distilled water and incubated with detection antibodies (1 μg/ml in PBS; clone XMG1.2, BD PharMingen) for 2–4 hours. After washing (6 washes in 200 μL/well PBS) and incubation with alkaline phosphatase conjugate (1:1000 dilution, Sigma; 1–2 hours at RT), colorimetric substrate (Sigma, in PBS) was used for visualization of separate dots. ELISPOT reader (BioReader 4000 Pro-X, BIO-SYS, Germany) was used to calculate the number of dots.

### Antigen-specific proliferative test

For proliferative test, cells (2–4×10^5^ cells/well) from popliteal lymph nodes were activated in the presence of antigens (10 μg/ml). As a control, APC and T-cells without antigen were used. After48 hours of incubation, 0.5 μKu of methyl-[3H]-timidine was added for 18 hours. Cells were transferred to the silica filters with semi-automated harvester (Scarton, Norway) and counted on liquid scintillator (Wallac, Finland).

### Secretion of IFNγ by lymphocytes of immunized animals in response to antigen activation

Splenocytes from immunized mice (5x10^6^ cells) were activated by antigen-vaccine components Ag85A, ESAT-6, CFP-10 (2 μg/ml), ultrasonicated (US) BCG lysate (10^8^ CFU/ml) or mitogen concavaline A (5 μg/ml, Sigma) as a positive control and PBS as a negative control. IFNγ level was estimated by ELISA (IFNγ kit, Bender MedSystems).

### Antigen-specific antibody titers

One week after the last immunization, sera were collected from each mouse and ESAT6, CFP-10 and Ag85a specific endpoint titers for IgG were detected by ELISA. Sera from the mice—treated with PBS and adjuvant alone—were used as a negative control. Antibody titers in each group were expressed as reciprocal end point titers by comparison with the PBS and BCG control with the value of P/N (Positive/Negative) >2.1. The results were expressed as the mean log10 endpoint titres per group (n = 3).

### TB modeling

#### Murine models

Six weeks after the last immunization, mice were challenged with virulent *M*. *Tuberculosis* H37Rv via the respiratory route or intravenously. For aerosol infection, frozen bacteria stocks were thawed and diluted to 10^6^ CFU/mL, and nebulized in an aerosol infection chamber (GLAS-COL APPARATUS CO 099C A4224) to get 10^3^ CFU per animal. For acute infection, bacteria diluted to 5x10^7^ CFU/mL were injected retro orbitally (100μL, PBS supplemented tween-80 0.05%). Infectious dose was determined by plating whole lung or spleen homogenates on day 30 after infection. Ten weeks later, the animals were euthanized, and lungs and spleens were homogenised in saline and plated at 10-fold serial dilutions on Middlebrook 7H11 agar (BD, NJ, USA) enriched with 10% FBS.

#### Guinea pig model

One week after last immunization, groups of 10 animals were aerosol challenged with 5×10^7^ CFU/mL bacteria in an aerosol infection chamber (GLAS-COL APPARATUS CO 099C A4224). Ten weeks later, the animals were euthanized, and their lungs and spleens were homogenised in saline and plated at 10-fold serial dilutions on Middlebrook 7H11 agar (BD, NJ, USA) enriched with 10% FBS. An independent group (10 animals) was observed for 120 days for vaccine protective effect evaluation.

### Statistical analysis

For all the quantitative data collected during experiments, sample means were calculated by averaging technical replicates. Sample means were used to calculate group means based on at least three independent sample values (n>3). Individual sample means were used to calculate inferential error bars for between-group comparisons on graphs. Both standard error of the mean (SEM) and 95% confidence intervals (95% CI) were calculated [23]. Kruskal-Wallis and Mann—Whitney U test were used for statistical analysis. Differences were considered as statistically significant at P<0.05. Data were statistically processed using STATISTICA, Microsoft Excel and GraphPad Prism 6. Log-Rank test was used for statistical analysis of both survival and illness curves. The value P<0.05 was considered to indicate a statistically significant result.

Results obtained were expressed on graphs as group means ±95% CI for error bars. Exact values of *n* for the experiments are stated in appropriate figure legends. Although graphs show results of single representative experiment, most of the animal experiments were repeated two or three times.

## Results

### Expression and purification of GamTBvac components and vaccine formulation

Antigens ESAT6, CFP10 and Ag85 are probably the most broadly recognized antigens for TB vaccine. They have been shown to be very immunogenic and immunodominant [13] and extensively used in several generations of recombinant subunit (H1/IC31, HyVac4 (H4)/IC31, H56/IC31, H56/CAF01 [24–30]), adenovirus [31,32] and live whole-cell [33] vaccines. Different expression/delivery strategies of these antigens showed different candidate vaccine effectiveness. In this study, fusions of known MTB antigens (ESAT6, CFP10 and Ag85) were combined with new adjuvant composition dextran/CpG. Fusions of MTB proteins (DBD-Ag85A and DBD-ESAT6-CFP10) cloned to expression vector (Fig 1A) were successfully expressed in *E*. *coli* (Fig 1B) and purified (Fig 1C). Western blotting with protein specific antibodies showed specific bands for both fusions (Fig 1D). Both fusions had expected sizes (DBD-ESAT6-CFP10–39.2 kDa and DBD-Ag85A – 48.6 kDa) except for overloading cases. The endotoxin level was less than 16 EU/mg, which was lower than the required limit for animal and *in vitro* experiment. The GamTBvac vaccine was prepared as a mix of antigens DBD-Ag85a and DBD-ESAT6-CFP10 (5, 10 or 20 μg/dose) with adjuvant containing dextran 500kDa (10 mg/dose), dextran-DEAE 500kDa (0.5 mg/dose) and CpG (0.15 mg/dose).

### GamTBvac showed strong immunogenicity in lymph nodes, spleen and lungs

#### Activation of IFNγ production in response to the presence of vaccine components

It is a widely accepted practice to choose vaccine antigens and adjust its concentrations for the T-cells of immunized mice to be able to induce IFNγ production—in response to their stimulation by the same antigen. In this paper, initial immunogenicity of vaccine components was studied by assessing activated T-cells IFNγ production level using the ELISPOT technique.

**Fig 2:**
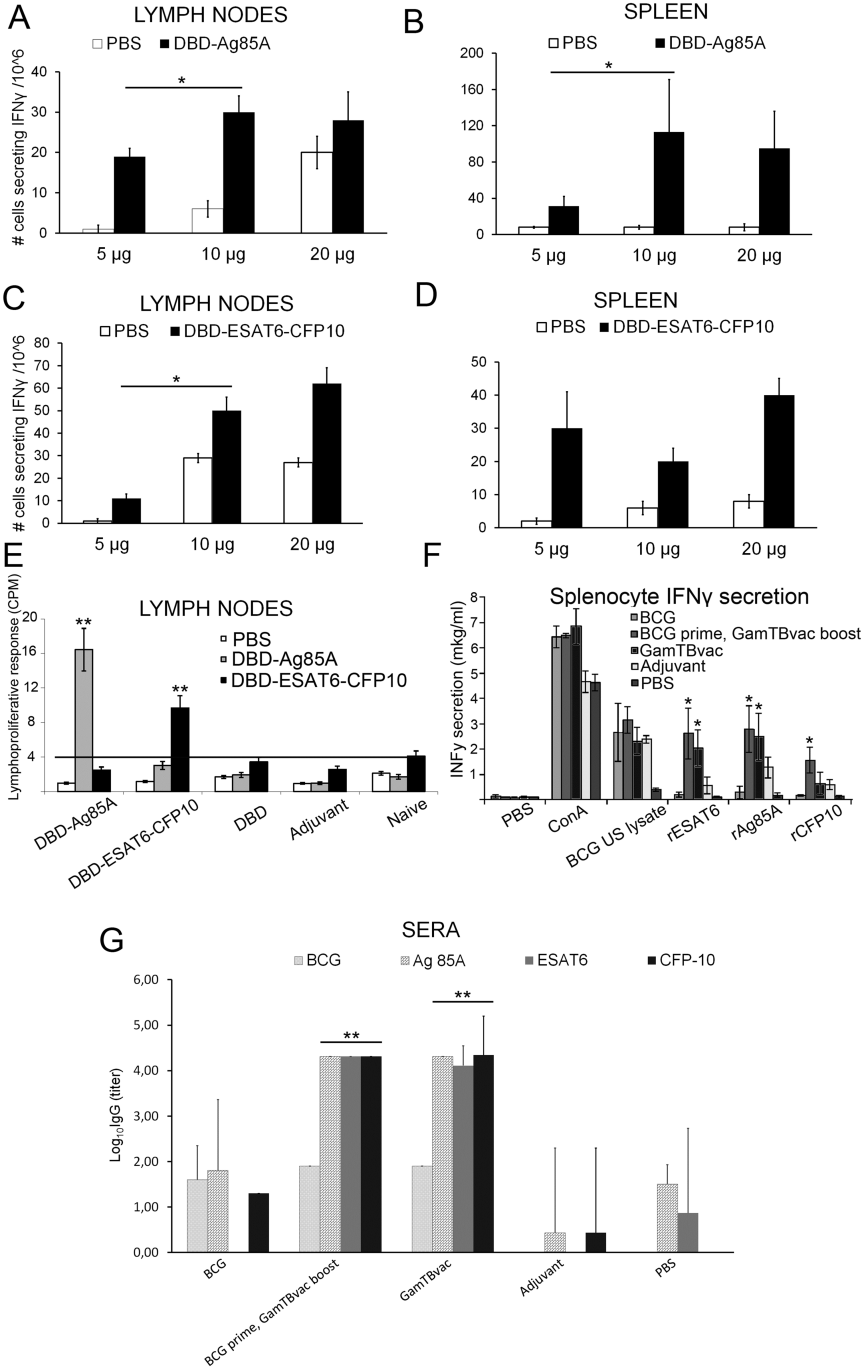
Immunogenicity of GamTBvac components in GamTBvac-prime/boost and BCG-prime/GamTBvac-boost regimens. The popliteal lymph node **(A, C)** and spleen **(B, D)** cells (0.5–8×10^5^/well) of the vaccinated mice were stimulated with DBD-Ag85A (10 μg/ml; **A, B**), DBD-ESAT6-CFP10 (10 μg/ml; **C, D**) separately *in vitro* and number of IFNγ secreting cells was assessed by ELISPOT. To estimate the lymphoproliferative potential of vaccine components, the popliteal lymph node cells of the vaccinated mice (2–4×10^5^ cells/well) were stimulated with DBD-Ag85A (10 μg/ml), DBD-ESAT6-CFP10 (10 μg/ml) or PBS separately *in vitro* (**E)** five weeks after the final immunization with specific antigen composition. Total IFNγ secretion in splenocytes upon different vaccination regimens was estimated by ELISA **(F)**. To evaluate the antigen-specific antibody titer, sera were collected from each mouse one week after last immunization. Endpoint IgG titers specific to ESAT6, CFP10 and Ag85a were detected by ELISA. Sera from mice treated with PBS or adjuvant (without antigen) were used as a negative control **(G)**. Results were presented as group means ± 95%CIs, n = 4, * P<0.05, ** P<0.01. In graphs F and C asterisk indicate significant differences compare to both PBS and BCG groups.

At the first stage of immunogenicity assessment, several antigen concentrations were tested. The C57Bl6 mice were immunized with 5, 10 or 20 μg of antigens—twice at three-week intervals in the presence of adjuvant. Five weeks after the last immunization, the number of IFNγ-secreting cells was counted using ELISPOT technique. Both antigens Ag85A and ESAT6-CFP10 induced IFNγ production in lymph nodes and spleen (Fig 2A–2C). For future experiments, 10 μg of each antigen was chosen as the smallest significantly (*P<0.05) efficient dose for both antigens in both lymph nodes and spleen (marked with asterisk). In the control group of the same experiment with single BCG immunization, 132 ± 12 cells produced IFNγ in spleen in response to MTB antigens extract. This response was comparable to Ag85A but significantly higher than ESAT6-CFP10 (*P<0.05). It should be noted that in separate series of experiments, DBD alone did not show significant effect on IFNγ induction by T-cells (data not shown). Thus, both antigens appear to be immunogenic when delivered with dextran/CpG adjuvant.

#### T-cell proliferation in the presence of vaccine components

Lymphoproliferative test was carried out to estimate exact contribution of each antigen (DBD-Ag85A, DBD-ESAT6-CFP10 or DBD) to immunogenicity of vaccine composition. The proliferative potential of lymphocytes in response to specific antigen stimulation was studied. For this experiment, immunization was carried out by separate GamTBvac antigens (DBD-Ag85A, DBD-ESAT6-CFP10 or DBD) in the presence of adjuvant or adjuvant without antigen. The general immunization protocol was the same as described above. Naïve group of mice was used as the control group. Popliteal lymph node cells were activated in the presence of 10 μg/ml of antigens (DBD-Ag85A or DBD-ESAT6-CFP10). Activation by DBD-Ag85A and DBD-ESAT6-CFP10 (Fig 2E) was significant (**P<0.01) compared to other groups only in the corresponding immunization groups (DBD-Ag85A and DBD-ESAT6-CFP10 respectively). In 66 hours after induction, proliferation activation was 7–10 times for DBD-Ag85A and 2–4 times for DBD-ESAT6-CFP10 over the background. Lymphoproliferative test completely matched with ELISPOT results. Ag85A and ESAT6-CFP10 are the very antigens responsible for specific immunogenicity but not the DBD domain alone or adjuvant components without antigens.

#### Immunogenicity assessment of GamTBvac components in GamTBvac-prime/boost and BCG-prime/GamTBvac-boost regimens

Most of the recombinant adjuvanted and viral TB vaccines were used as the boosters for initial BCG vaccination [24–32]. So, it was important to study immunogenicity from the beginning with regards to the immunization scheme including booster BCG component. To study the contribution of separate GamTBvac antigens to the immunity gained by GamTBvac in context of different vaccination schemes, separate recombinant antigens (rAg85A, rESAT6 and rCFP10) were used as IFNγ inducers in splenocytes. IFNγ concentration was measured by ELISA in groups of mice immunized with BCG, BCG-prime/ GamTBvac-boost, GamTBvac-prime/boost regimens (Fig 2F). PBS, Concanavalin A (ConA) and BCG ultrasonicated lysate were used as controls. Ag85A and ESAT6 gave the strongest and significant (*P<0.05) induction of IFNγ production in both BCG-prime/GamTBvac-boost and GamTBvac-prime/boost regimens compare to PBS and BCG groups. It is worth noting that CFP10 significantly (*P<0.05) induced IFNγ production only in BCG-prime/GamTBvac-boost regimen but not in GamTBvac-prime/boost regimen. Activation in ConA and absence of activation in PBS groups indicate that the experimental setup was correct. BCG lysate showed IFNγ activation in all experimental groups due to non-specific IFNγ induction factors present in BCG lysate. To sum up, all antigens (Ag85A, ESAT6 and CFP10) contribute to GamTBvac immunogenicity. Immunogenicity of all antigens is specifically indicated for BCG-prime/GamTBvac-boost regimen.

#### Antigen-specific antibody titers

The role of antibodies in protection against MTB is not clear. Poor induction of humoral responses by BCG leads to the assumption that antibodies play no role in TB immunity. Some authors [4] debate this point of view. It seems that antibody production is at least an indirect indicator of immunogenicity mounted by TB vaccine candidates, while the broader role of antibody may be in providing immunity to aerosol MTB and sterile infection [4]. Thus, induction of antibody response needs to be taken into account in every TB vaccine candidate.

ELISA estimation of antigen specific antibody titers was carried out in groups of mice immunized with BCG, BCG-prime/GamTBvac-boost and GamTBvac-prime/boost regimens. PBS, adjuvant-alone and BCG did not induce significant increase of antibody titers. Significantly higher antibody titers against Ag85A, ESAT-6 and CFP10 versus both PBS and BCG groups (**P<0.01) were observed only for BCG-prime/GamTBvac-boost and GamTBvac-prime/boost vaccination regimens (Fig 2G).

### Protective efficacy of GamTBvac

#### Protective efficacy of GamTBvac and its components in TB murine models

**Fig 3:**
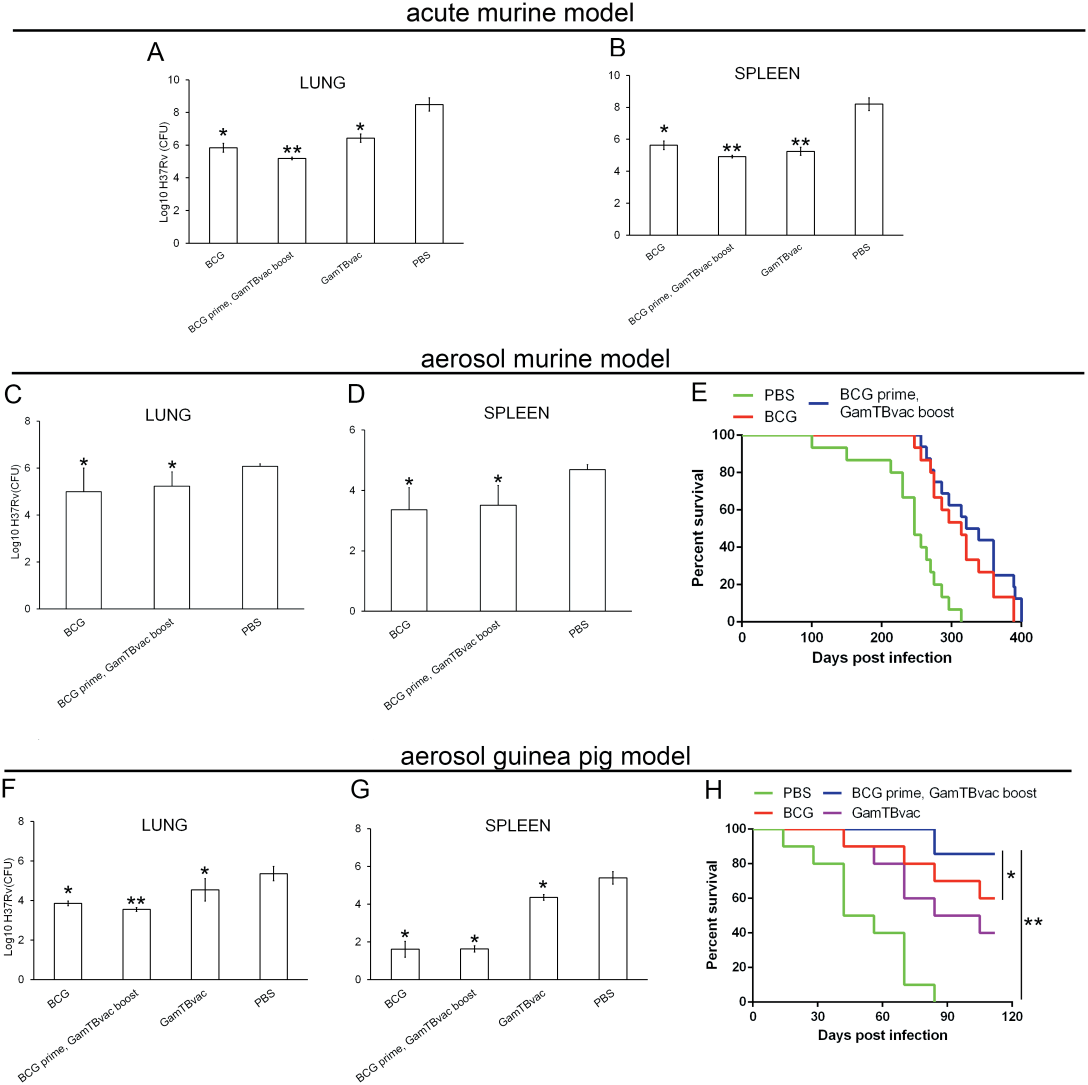
Protective efficacy of GamTBvac against *M*. *tuberculosis* H37Rv infection in murine and guinea pig models. The mice were immunized subcutaneously with GamTBvac and BCG (5×10^6^ CFU) in different regimens. BCG was administered once at the 1^st^ week and GamTBvac twice (three weeks apart). The control group was injected with PBS. For the acute murine model, the mice were retro-orbitally infected with MTB H37Rv two weeks after the last immunization. The results presented are given as mean Log_10_ CFU per organ ± 95%CI from groups of seven mice (n = 7), * P<0.05, relative to PBS; ** P<0.05, relative to both PBS and BCG. Thirty days after infection, protective efficacy was measured (n = 6) in lungs **(A)** and spleens **(B)**. For the aerosol murine model, the mice were infected in aerosol infection chamber to get 10^3^ CFU per animal sixty days after the last immunization. Four weeks later, bacterial load (n = 6) was determined in lungs **(C)** and spleens **(D)**. Aerosol infection (n = 15) was monitored for more than 400 days until all mice died **(E)**.

The protective efficacy of a vaccine is characterized by its capacity to protect animals from death and reduce mycobacteria growth in the organs of the infected animals. Two infectious models—murine and guinea pig—were used to assess protective efficacy. For acute murine model, four mice groups (C57Bl6 line) were immunized with BCG, BCG-prime/GamTBvac-boost, GamTBvac-prime/boost or PBS as negative control. The mice were intravenously infected with MTB H37Rv strain (8.3×10^6^ in 0.1 ml) two weeks after the last immunization in all experimental groups. After 30 days, six mice from each experimental group were used for assessment of bacteria load in lungs and spleen (Fig 3A and 3B).

Guinea pigs were subcutaneously immunized with BCG (5×10^6^ CFU) and/or GamTBvac (10 μg of each recombinant component DBD-Ag85A, DBD-ESAT6-CFP10) formulated with adjuvant in different regimens. BCG was administered once at the 1^st^ week and GamTBvac twice (5^th^ and 8^th^ weeks). The control group was injected with PBS. Two weeks after the last vaccination, the animals were aerosol-infected with MTB H37Rv. Six weeks later, the protective efficacy in lungs **(F)** and spleens **(G)** was measured. The survival curve **(H)** depicts the experiment where guinea pigs were monitored for 120 days (n = 10, * P<0.05 vs PBS, ** P<0.01 vs BCG).

All experimental groups showed that mycobacterial lung load (CFU) (Fig 3A) and mycobacterial spleen load (Fig 3B) were 2–3 orders of magnitude lower than the control group. GamTBvac vaccination showed significantly lower titer than the BCG group in spleen but not in lungs. The most prominent difference was observed in those mice vaccinated with BCG-prime/GamTBvac-boost regimen compared to BCG control.

The experimental groups were followed for two months after infection. The number of mice that died after infection was almost the same with the experimental groups, but was lower than the control group—11.1–10.5% in the experimental groups and 55.5% in the control (data not shown). However, the average length of survival in the group immunized with BCG vaccine was only 17 days compare to 24 days in the control. The average length of survival in groups immunized with GamTBvac in both prime and boost regimens was about 50 days, which indicates the efficacy of recombinant vaccine. Nonetheless, the infection achieved in acute murine model is extremely aggressive. The bacterial load in unvaccinated mice reached almost 10^9^ bacteria in lungs—this translates into over 50% mortality and hence the model has limited relevance from a clinical standpoint. So, we set up murine and guinea pig aerosol infection models.

Aerosol infection of mice four weeks after the last immunization showed that BCG immunization reduced mycobacterial titer by more than one order of magnitude in lungs and spleen but boost vaccination with GamTBvac did not show significant additional effect on bacterial load in lungs (Fig 3C) and spleens (Fig 3D). Further monitoring of infected animals showed that both BCG and BCG-prime/GamTBvac-boost significantly reduced death rate (P = 0.042 and P = 0.0382 respectively). Nevertheless, GamTBvac as a BCG booster did not improve results compare to BCG group (P = 0.99) (Fig 3E).

These experiments show that GamTBvac can deliver results similar to that of BCG in some endpoints and carry additional efficacy as a BCG booster. It is a known that murine TB model has many limitations and that guinea pigs may be more acceptable for TB infection modeling [9]. Therefore, the next series of experiments were carried out in an aerosol guinea pig TB model.

#### Protective efficacy of GamTBvac and its components on aerosol guinea pig model

Four experimental groups of 8 animals each were immunized with BCG, BCG-prime/GamTBvac-boost, GamTBvac-prime/boost and PBS. BCG was injected only once, whereas GamTBvac was subcutaneously injected twice on the 5^th^ and 8^th^ week in the relevant groups. Thirty days after aerosol infection, the GamTBvac-prime/boost group had one order of magnitude less mycobacteria in lungs (Fig 3F) and spleen (Fig 3G) but over one order of magnitude more CFU than in the BCG control group. The BCG-prime/GamTBvac-boost group again showed the best result in lungs with more than half an order of magnitude less mycobacteria in both lungs and spleen. It is important that BCG-prime/GamTBvac-boost group (Fig 3F) showed significantly less mycobacterial load in lungs compare to BCG group (**P<0.05).

For survival analysis, a separate experiment with the same experimental groups (10 animals each) was carried out (Fig 3H). Ninety percent of animals in the BCG-prime/GamTBvac-boost group were alive 110 days after infection. This was significantly more than 60% in BCG-prime group (P = 0.0391) and PBS control group (p = 0.00911) where all the animals died 85 days after infection. In the GamTBvac-prime/boost group, 40% of animals were alive.

## Discussion

Studies on the efficacy of BCG vaccination showed significant latitude-dependent gradient on high efficacy in Northern Europe and North America—to almost absence of protection in the southern regions close to the equator [34]. BCG protection lasts for at least 15 years [35] but some reports suggest that it could be much longer [8,36]. It is believed that MTB infection and environmental mycobacteria sensitization reduces BCG effectiveness against pulmonary and possibly miliary and meningeal tuberculosis. Detailed mechanism is unknown but it is clear that new vaccines and delivery strategies should take this fact into account.

Live mycobacterial vaccines such as recombinant BCG or attenuated MTB are intended as replacements for neonatal BCG vaccination. However, recent studies show that expression of MTB antigens on such platforms could be unsafe [37]. Being highly immunogenic and capable of inducing bactericidal activity in blood, the AERAS-442 vaccine reactivated varicella zoster virus (VZV) in two cases of clinical trials. The reasons for VZV reactivation is not known but the MTB antigens (Ag85A, Ag85B or Rv3407) used in the study are unlikely to be the cause. Recombinant protein vaccines were generally considered safer, but the protection given by such vaccines lasts for a shorter time [4].

In this study, very well-known TB antigens (ESAT6, CFP10 and Ag85), extensively used as vaccine components [24–33], were combined with the novel dextran/CpG adjuvant. Mixture of dextran and dextran-DEAE allows depositing both antigens fused with DBD and CpG composition through relatively week electrostatic interaction. This multicomponent adjuvant plays the role of an antigen depo and allows stimulation of TLR9 by CpG (ODN 2216 ggGGGACGA:TCGTCgggggg [38]) and activation of phagocytosis through interaction between dextran and DC-SIGN receptors [39]. Thus, the complex dextran/CpG adjuvant allows for slow antigen release (our unpublished data) and Th1 polarisation of immune response.

Immunogenicity experiments indicate that GamTBvac can induce both humoral and cellular immune responses (Fig 2). MTB antigens (Ag85A, ESAT-6 and CFP10)–included in the candidate vaccine—induce specific IFNγ production by cells circulating through the lymph nodes and spleen (Fig 2A–2D). Lymph node cells proliferate efficiently (Fig 2E) in response to Ag85A and ESAT6-CFP10. These very antigens are responsible for specific immunogenicity, not the DBD domain alone or adjuvant components without antigens. According to the present data all antigens (Ag85A, ESAT6 and CFP10) contribute to the immunogenicity of GamTBvac. The immunogenicity of all antigens is specifically indicated for BCG-prime/GamTBvac-boost regimen (Fig 2F). It is unclear why CFP10 induces IFNγ only when GamTBvac is used as a booster. GamTBvac induces particularly strong specific antibody response against selected antigens (Ag85A, ESAT6 and CFP10) in both GamTBvac-prime/boost and BCG-prime/GamTBvac-boost regimens. The humoral immunity mounted by GamTBvac could additionally improve the protective power of candidate TB vaccine. Thus, different lines of evidence indicate that GamTBvac is immunogenic and is a good candidate to test the protective properties of antigens.

Animal models are another big problem in TB vaccine development. Animal models do not reflect human infection [4]. Besides, it is hard to reproduce the physiological method of infection in humans on animals. Nevertheless, it was learnt from MVA85A vaccine [40] that protection in animal models is an important indicator and can predict efficacy in human trials [41]. Importantly, GamTBvac showed protective immunity in both murine and guinea pig TB infection models. Indeed, in both models the strongest protection was provided by BCG-prime/GamTBvac-boost regimen with complementary protection to BCG (Fig 3). In general, experiments presented in this paper suggest that GamTBvac candidate vaccine could be efficient as a BCG booster. This allowed us to proceed with GamTBvac for clinical trials (reg.№179 from 10.04.2015 http://grls.rosminzdrav.ru/).

Recombinant TB vaccine candidates now show competitive results compared to BCG administered in BCG-prime recombinant vaccine-boost [9,14,30] and even recombinant vaccine-prime [15] regimens. In animal TB models, BCG works quite well, while long-term experiments are limited by the life span of experimental animals. It is especially hard to compete with BCG during early days after vaccination. Thus, the additive effect of recombinant vaccine in BCG-prime regimen is an important indicator for evaluation of a vaccine candidate. A prolonged time (up to 30 weeks) between the last vaccination and infection challenge [15] could be used to evaluate the efficacy of a vaccine. Such approach might highlight the long-term effect of recombinant vaccine against the backdrop of the fading BCG-mounted protective immunity.

There is concern that GamTBvac can interfere with IFNγ release assays (IGRA) for detection of LTBI, such as QFT, because ESAT6 and CFP10 are the main components of IGRA tests. This issue is relevant for a wide range of vaccine candidates using ESAT6 and CFP10 as antigens [24–32]. As recently reported, H1/IC31 vaccination can lead to conversion of the QFT result because of its ESAT-6 component in one third of initially QFT negative participants [26,42,43]. However, reversions suggest that some of the observations might also be attributed to the within-subject variability of the QFT assay [44]. A GamTBvac clinical study will reveal the precise QFT conversion rate. The World Health Organization recommend that IGRA should not replace tuberculin skin tests in resource-constrained settings [1], where the vaccine would be broadly used for the first time. However, new tests using diagnostic markers other than ESAT-6 and CFP10 for disease screening are being developed and have the potential to in the future eliminate the issue of interference with vaccines containing these antigens [45,46].

## References

[1] WHO, Global Tuberculosis Report 2016. 21^th^ edition France: World Health Organisation; 2016;214

[2] Andersen P, Urdahl KB. TB vaccines; promoting rapid and durable protection in the lung. Curr Opin Immunol. Elsevier Ltd; 2015;35: 55–62. 10.1016/j.coi.2015.06.001 PMC4641675 26113434

[3] Glaziou P, Sismanidis C, Floyd K, Raviglione M. Global Epidemiology of Tuberculosis. Cold Spring harb Perspec Med. 2015; 1–18. 10.1101/cshperspect.a017798 PMC4315920 25359550

[4] Karp CL, Wilson CB, Stuart LM. Tuberculosis vaccines: Barriers and prospects on the quest for a transformative tool. Immunol Rev. 2015;264: 363–381. 10.1111/imr.12270 25703572 PMC4368410

[5] Andersen P, Doherty TM. The success and failure of BCG—implications for a novel tuberculosis vaccine. Nature reviews Microbiology. 2005; 3(8):656–62. 10.1038/nrmicro1211 16012514

[6] Pang Y, Zhao A, Cohen C, Kang W, Lu J, Wang G, et al Current status of new tuberculosis vaccine in children. Hum Vaccin Immunother. 2016;5515: 1–11. 10.1080/21645515.2015.1120393 PMC4962963 27002369

[7] Fine PE Variation in protection by BCG: implications of and for heterologous immunity. Lancet 1995;346: 1339–1345. S0140-6736(95)92348-9. 7475776 10.1016/s0140-6736(95)92348-9

[8] Nguipdop-Djomo P., Heldal E., Rodrigues L. C., Abubakar I., & Mangtani P. Duration of BCG protection against tuberculosis and change in effectiveness with time since vaccination in Norway: A retrospective population-based cohort study. The Lancet Infectious Diseases 2016;16(2): 219–226. 10.1016/S1473-3099(15)00400-4 26603173

[9] Skeiky YAW, Dietrich J, Lasco TM, Stagliano K, Dheenadhayalan V, Goetz MA, et al Non-clinical efficacy and safety of HyVac4:IC31 vaccine administered in a BCG prime-boost regimen. Vaccine. 2010;28: 1084–1093. 10.1016/j.vaccine.2009.10.114 19896449

[10] Rodrigues LC, et al Effect of BCG revaccination on incidence of tuberculosis in school-aged children in Brazil: the BCG-REVAC cluster-randomised trial. Lancet 2005;366(9493):1290–5. 10.1016/S0140-6736(05)67145-0 16214599

[11] Frick M. The Tuberculosis Vaccines Pipeline: A New Path to the Same Destination? HIV, HCV & TB Pipeline Report (Treatment Action Group). HIV& TB (2015). July 2015. http://www.pipelinereport.org/2015/tb-vaccines. Cited 25 August 2016.

[12] Agger EM. Novel adjuvant formulations for delivery of anti-tuberculosis vaccine candidates. Adv Drug Deliv Rev. Elsevier B.V.; 2015; 10.1016/j.addr.2015.11.012 PMC4870161 26596558

[13] Carpenter C, Sidney J, Kolla R, Nayak K, Tomiyama H, Tomiyama C, et al A side-by-side comparison of T cell reactivity to fifty-nine Mycobacterium tuberculosis antigens in diverse populations from five continents. Tuberculosis. Elsevier Ltd; 2015;95: 713–721. 10.1016/j.tube.2015.07.001 PMC4666753 26277695

[14] Xin Q, Niu H, Li Z, Zhang G, Hu L, Wang B, et al Subunit Vaccine Consisting of Multi-Stage Antigens Has High Protective Efficacy against Mycobacterium tuberculosis Infection in Mice. PLoS One. 2013;8: 1–12. 10.1371/journal.pone.0072745 PMC3744459 23967337

[15] Niu H, Peng J, Bai C, Liu X, Hu L, Luo Y, et al Multi-stage tuberculosis subunit vaccine candidate LT69 provides high protection against Mycobacterium tuberculosis infection in mice. PLoS One. 2015;10: 1–11. 10.1371/journal.pone.0130641 PMC4476732 26098302

[16] Baldwin SL, Bertholet S, Reese VA, Ching LK, Reed SG, Coler RN. The importance of adjuvant formulation in the development of a TB vaccine. J Immunol. 2012;188: 2189–2197. 10.4049/jimmunol.1102696 22291184 PMC3288309

[17] Tukhvatulin AI, Dzharullaeva AS, Tukhvatulina NM, Shcheblyakov D V., Shmarov MM, Dolzhikova I V., et al Powerful Complex Immunoadjuvant Based on Synergistic Effect of Combined TLR4 and NOD2 Activation Significantly Enhances Magnitude of Humoral and Cellular Adaptive Immune Responses. PLoS One. 2016;11: e0155650 10.1371/journal.pone.0155650 27187797 PMC4871337

[18] Knudsen NPH, Olsen A, Buonsanti C, Follmann F, Zhang Y, Coler RN, et al Different human vaccine adjuvants promote distinct antigen- independent immunological signatures tailored to different pathogens. 2016; 1–13. 10.1038/srep19570 PMC4726129 26791076

[19] Cordeiro AS, Alonso MJ, de la Fuente M. Nanoengineering of vaccines using natural polysaccharides. Biotechnol Adv. Elsevier B.V.; 2015;33: 1279–1293. 10.1016/j.biotechadv.2015.05.010 PMC7127432 26049133

[20] Cox J.C., Coulter A.R. Adjuvant—a classification and rewiew of their modes of action. Vaccine. 1997;15 (3) 248–256. 9139482 10.1016/s0264-410x(96)00183-1

[21] Ershova AS, Gra OA, Lyaschuk AM, Grunina TM, Tkachuk AP, Bartov MS, et al Recombinant domains III of Tick-Borne Encephalitis Virus envelope protein in combination with dextran and CpGs induce immune response and partial protectiveness against TBE virus infection in mice. BMC Infect Dis. 2016;16: 544 10.1186/s12879-016-1884-5 27717318 PMC5054610

[22] Tkachuk A., Lunin V., Karyagina A., Gintsburg A. Problems and prospects of development of the subunit TB vaccine. J Acquir Immune Defic Syndr 2014; 65(Suppl 2): 43.

[23] Cumming G, Fidler F, Vaux DL. Error bars in experimental biology. J Cell Biol. 2007;177: 7–11. 10.1083/jcb.200611141 17420288 PMC2064100

[24] Olsen AW, Pinxteren L, Okkels LM, Rasmussen PB, Andersen P. Protection of Mice with a Tuberculosis Subunit Vaccine Based on a Fusion Protein of Antigen 85B and ESAT-6. Microbiology. 2001;69: 2773–2778. 10.1128/IAI.69.5.2773-2778.2001 PMC98224 11292688

[25] Agger EM, Rosenkrands I, Olsen AW, Hatch G, Williams A, Kritsch C, et al Protective immunity to tuberculosis with Ag85B-ESAT-6 in a synthetic cationic adjuvant system IC31. Vaccine. 2006;24: 5452–5460. 10.1016/j.vaccine.2006.03.072 16675077

[26] Reither K, Katsoulis L, Beattie T, Gardiner N, Lenz N, Said K, et al Safety and immunogenicity of h1/ic31h, an adjuvanted tb subunit vaccine, in hiv-infected adults with cd4+ lymphocyte counts greater than 350 cells/mm3: A phase ii, multi-centre, double-blind, randomized, placebo-controlled trial e114602. PLoS One. 2014;9: 1–19. 10.1371/journal.pone.0114602 PMC4260867 25490675

[27] Geldenhuys H, Mearns H, Miles DJC, Tameris M, Hokey D, Shi Z, et al The tuberculosis vaccine H4: IC31 is safe and induces a persistent polyfunctional CD4 T cell response in South African adults: A randomized controlled trial. Vaccine. Elsevier Ltd; 2015;33: 3592–3599. 10.1016/j.vaccine.2015.05.036 26048780

[28] Aagaard C, Hoang T, Dietrich J, Cardona P-J, Izzo A, Dolganov G, et al A multistage tuberculosis vaccine that confers efficient protection before and after exposure. Nat Med. Nature Publishing Group; 2011;17: 189–194. 10.1038/nm.2285 21258338

[29] Billeskov R, Elvang TT, Andersen PL, Dietrich J. The HyVac4 subunit vaccine efficiently boosts BCG-primed anti-mycobacterial protective immunity. PLoS One. 2012;7. 10.1371/journal.pone.0039909 PMC3386939 22768165

[30] Woodworth JS, Cohen SB, Moguche AO, Plumlee CR, Agger EM, Urdahl KB, et al Subunit vaccine H56/CAF01 induces a population of circulating CD4 T cells that traffic into the Mycobacterium tuberculosis-infected lung. Mucosal Immunol. Nature Publishing Group; 2016; 10.1038/mi.2016.70 PMC5325828 27554293

[31] Li W, Li M, Deng G, Zhao L, Liu X, Wang Y. Prime-boost vaccination with Bacillus Calmette Guerin and a recombinant adenovirus co-expressing CFP10, ESAT6, Ag85A and Ag85B of Mycobacterium tuberculosis induces robust antigen-specific immune responses in mice. Mol Med Rep. 2015;12: 3073–3080. 10.3892/mmr.2015.3770 25962477

[32] Chen YY, Lin CW, Huang WF, Chang JR, Su IJ, Hsu CH, et al Recombinant bacille Calmette-Guerin coexpressing Ag85b, CFP10, and interleukin-12 elicits effective protection against Mycobacterium tuberculosis. J Microbiol Immunol Infect. Elsevier Taiwan LLC; 2014; 1–7. 10.1016/j.jmii.2014.11.019 25732698

[33] Hoft DF, Blazevic A, Selimovic A, Turan A, Tennant J, Abate G, et al Safety and Immunogenicity of the Recombinant BCG Vaccine AERAS-422 in Healthy BCG-naïve Adults: A Randomized, Active-controlled, First-in-human Phase 1 Trial. EBioMedicine. The Authors; 2016;7: 278–286. 10.1016/j.ebiom.2016.04.010 PMC4909487 27322481

[34] Mangtani P, Abubakar I, Ariti C, Beynon R, Pimpin L, Fine PEM, et al Protection by BCG vaccine against tuberculosis: A systematic review of randomized controlled trials. Clin Infect Dis. 2014;58: 470–480. 10.1093/cid/cit790 24336911

[35] Abubakar I, Pimpin L, Ariti C, Beynon R, Mangtani P, Sterne J, et al Systematic review and meta-analysis of the current evidence on the duration of protection by bacillus Calmette-Gue rin vaccination against tuberculosis. Health Technol Assess (Rockv). 2013;17: 1–4. 10.3310/hta17370 PMC4781620 24021245

[36] Aronson NE, Santosham Mathuram, Comstock GW, Howard RS, Moulton LH, Everett R. Rhoades, et al Long-term Efficacy of BCG Vaccine. 2004;291: 2086–2091. 10.1001/jama.291.17.2086 15126436

[37] Hoft DF, Blazevic A, Selimovic A, Turan A, Tennant J, Abate G, et al Safety and Immunogenicity of the Recombinant BCG Vaccine AERAS-422 in Healthy BCG-naïve Adults: A Randomized, Active-controlled, First-in-human Phase 1 Trial. EBioMedicine. The Authors; 2016;7: 278–286. 10.1016/j.ebiom.2016.04.010 PMC4909487 27322481

[38] Mohamed W, Domann E, Chakraborty T, Mannala G, Lips KS, Heiss C, et al TLR9 mediates S. aureus killing inside osteoblasts via induction of oxidative stress. BMC Microbiol. BMC Microbiology; 2016; 1–8. 27716055 10.1186/s12866-016-0855-8 PMC5048406

[39] Pustylnikov S, Sagar D, Jain P, Khan ZK. Targeting the C-type Lectins-Mediated Host-Pathogen Interactions with Dextran. 2014;17: 371–392. 10.18433/j3n590 PMC5553543 25224349

[40] Tameris MD, Hatherill M, Landry BS, Scriba TJ, Snowden MA, Lockhart S, Shea JE, McClain JB, Hussey GD, Hanekom WA et al: Safety and efficacy of MVA85A, a new tuberculosis vaccine, in infants previously vaccinated with BCG: a randomised, placebo-controlled phase 2b trial. Lancet 2013;381:1021–1028. 10.1016/S0140-6736(13)60177-4 23391465 PMC5424647

[41] Beverley P: TB vaccine failure was predictable. Nature. 2013;503:p469; 10.1038/503469e 24284721

[42] van Dissel JT, Arend SM, Prins C, Bang P, Tingskov PN, Lingnau K, et al Ag85B-ESAT-6 adjuvanted with IC31 promotes strong and long-lived Mycobacterium tuberculosis specific T cell responses in naive human volunteers. Vaccine. 2010;28: 3571–3581. 10.1016/j.vaccine.2010.02.094 20226890

[43] van Dissel JT, Soonawala D, Joosten SA, Prins C, Arend SM, Bang P, et al Ag85B-ESAT-6 adjuvanted with IC31 promotes strong and long-lived Mycobacterium tuberculosis specific T cell responses in volunteers with previous BCG vaccination or tuberculosis infection. Vaccine. Elsevier Ltd; 2011;29: 2100–2109. 10.1016/j.vaccine.2010.12.135 21256189

[44] Van Zyl-Smit RN, Pai M, Peprah K, Meldau R, Kieck J, et al Within-subject variability and boosting of T-cell interferon-gamma responses after tuberculin skin testing. Am J Respir Crit Care Med 2009;180: 49–58. 10.1164/rccm.200811-1704OC 19342414

[45] Frick B. M. The Tuberculosis Prevention Pipeline. 2016:143–162.

[46] Millington KA, Fortune SM, Low J, Garces A, Hingley-Wilson SM, et al Rv3615c is a highly immunodominant RD1 (Region of Difference 1)-dependent secreted antigen specific for Mycobacterium tuberculosis infection. Proc Natl Acad Sci U S A 2011;108: 5730–5735. 10.1073/pnas.1015153108 21427227 PMC3078386

